# A Feeling of Otherness: A Qualitative Research Synthesis Exploring the Lived Experiences of Stigma in Individuals with Inflammatory Bowel Disease

**DOI:** 10.3390/ijerph18158038

**Published:** 2021-07-29

**Authors:** Kate Muse, Emma Johnson, Annabel L. David

**Affiliations:** 1 School of Psychology, University of Worcester, Worcester WR2 6AJ, UK; 2 Children’s Psychological Medicine, Oxford Children’s Hospital, Oxford University Hospitals NHS Foundation Trust, Oxford OX3 9DU, UK; emma.johnson10@outlook.com (E.J.); Annabel.David@ouh.nhs.uk (A.L.D.)

**Keywords:** stigma, inflammatory bowel disease, colitis, ulcerative, Crohn’s disease, meta-synthesis, patient experiences, qualitative research

## Abstract

Inflammatory bowel disease (IBD) consists of Crohn’s disease and ulcerative colitis, chronic conditions involving inflammation and ulceration of the gastrointestinal tract. Individuals with IBD may be susceptible to experiencing health-related stigma: experienced, perceived, or internalised social exclusion, rejection, blame, or devaluation resulting from negative social judgements based on the disease. This qualitative research synthesis draws together findings from 38 studies describing lived experiences to develop a unified interpretative account of the experience of stigma in IBD. Analysis developed two categories: ‘The IBD journey’ explores the dynamic ways in which having IBD impacted on individuals’ self-identity and ‘a need to be understood’ examines the tension between wanting to be understood whilst feeling their true experiences needed to be hidden from or were misjudged by the social sphere. The overarching concept ‘feeling of otherness’ highlights that, rather than a static, binary experience, individuals moved across a continuum ranging from the excluding experience of feeling stigmatised and othered, to the inclusive experience of integration. Individuals fluctuated along this continuum across different physical, social, and health contexts. Psychological adjustment to IBD, drawing on experience of adaptive coping, and reconnecting with valued others through illness disclosure strengthened stigma resistance during more challenging times.

## 1. Introduction

Inflammatory bowel disease (IBD) consists of Crohn’s disease and ulcerative colitis, chronic conditions involving inflammation and ulceration of the gastrointestinal tract. Symptoms include increased frequency and urgency to defecate, severe abdominal pain, blood in stools, weight loss, fatigue, and in some cases joint swelling/pain, eye inflammation, delayed puberty, growth delay, and perianal disease [1]. Individuals often experience periods of active symptoms or ‘flare-ups’ of severe symptoms and periods of remission [2]. IBD has accelerated in prevalence over recent years worldwide, surpassing 0.3% of the population in North America and Europe [3]. Although there is currently no cure for IBD, immunosuppressants, corticosteroids, and biological therapy can produce periods of symptom-free remission, though the medications themselves have side effects, such as increased appetite and weight gain, acne, increased risk of infections, low mood, anxiety, irritability, upper respiratory tract infections, headaches, abdominal pain, and nausea [4,5]. For those with complex or treatment-resistant presentation, surgical intervention may be required [1].

IBD can lead to a complex interplay of physical and psychological consequences. The psychosocial impacts of IBD are substantial and can be severely debilitating [6]. These include reduced quality of life, impaired interpersonal relationships, and increased prevalence of depression and anxiety [7,8,9,10]. It has long been suggested that stress, which can be caused both directly by IBD and indirectly by the additional challenges and burden it places on individuals, adversely affects the course of IBD by increasing disease activity [11,12]. Patients also report that their disease worsens when they feel anxious or depressed [13]. Stigma may contribute to, or further exacerbate, the complex physical, psychological, and social burden of IBD [14]. It is therefore important to better understand the role of stigma as a psychological process within the IBD illness experience, as this may help to improve objective and subjective indicators of the disease.

Stigma is a social construction that involves de-valuing an individual or group based on an attribute that characterises them as different [15]. It is exemplified by a convergence of labelling, cultural beliefs, disconnection, status loss, and discrimination [16]. People may experience stigma in relation to a number of enduring features of their identity, including their health. Weiss [14] offers a focussed definition of health-related stigma as a process of experienced, perceived, or anticipated social exclusion, rejection, blame, or devaluation resulting from a medically unwarranted adverse social judgement about an individual or group on the basis of identification with a particular health condition. Health-related stigma is prevalent across a number of chronic health conditions, most notably mental health, HIV/AIDS, and leprosy, but also in diabetes, asthma, epilepsy, multiple sclerosis, pain disorder, fibromyalgia, chronic fatigue syndrome, and irritable bowel syndrome [17,18]. Health-related stigma is also highly prevalent in people with IBD [19,20]. As the nature and impact of stigma is likely to vary across different health conditions [14], it is important to understand this phenomenon within the context of IBD.

Factors around disruptiveness, aesthetic qualities, concealability, disease course, origin, and danger, have been suggested as making conditions susceptible to health-related stigma [21] and may be applicable within IBD. Lack of awareness and understanding about the level of disability and chronicity of the disease, its cause, and the often hidden symptoms can result in individuals not being believed or being blamed for their condition [20]. IBD is a disease impacting the bowels, a taboo topic which is often viewed with disgust at a societal level [22]. Although a generally unseen condition, unpredictable ‘flare-ups’ can reveal noticeable, aesthetically displeasing, and disruptive symptoms or behavioural consequences, for example increased frequency and urgency to defecate resulting in increased toilet use and disruption to activities or social interactions. Some symptoms may be viewed as socially undesirable because they do not conform to sociocultural rules and expectations about bowel control and the private nature of bowel movements and faeces [23,24].

Stigma is multi-faceted, involving three key domains: enacted (direct experience of social discrimination), perceived (how much individuals feel others hold stigmatising beliefs or attitudes toward them), and internalised (the belief that negative attitudes or stereotypes are deserved, accurate and apply to oneself), [17]. Enacted stigma has not been well explored in IBD. However, a small number of studies found that individuals report being treated differently by others within work-related contexts [19,20]. Perceived stigma was more commonly reported in IBD, with individuals describing that family, friends, co-workers, teachers, healthcare professionals, and the broader community do not understand or take their condition seriously [19,20]. This may suggest that individuals do not commonly experience direct enactment of the stigmatising beliefs or attitudes they perceive others to hold, though it is difficult to draw clear conclusions due to the dearth of literature exploring enacted stigma. Mild levels of internalised stigma involving feeling unclean and damaged were reported by individuals with IBD, particularly by those in an active disease phase [19,20]. Stigma has been found to have a negative impact on a range of physical and mental health outcomes in IBD, including quality of life, psychological wellbeing, self-esteem and self-efficacy, health-seeking behaviour, treatment adherence, and relationships with healthcare professionals [19,20,25]. Whilst the perception that others hold negative views alone can reduce well-being, internalised stigma may result in the poorest psychological outcomes [20].

More recently stigma resistance has also been examined, with some individuals showing an adaptive response involving the ability to adjust to and even flourish in the face of adverse stigma experiences [25]. High levels of resistance to stigma have been reported in IBD, indicating that many individuals do not internalise the stigmatising attitudes they perceive others to hold into their own identity [20]. Although not yet well understood, social support, length of illness, public awareness, extraintestinal symptoms, educational level, and urban dwelling have been suggested as influencing stigma resistance [19].

Whilst a growing body of research has begun to document the prevalence and impact of stigma in IBD, the process of when, how, and why different forms of stigma impact these individuals is less well understood. A fuller understanding of the process of health-related stigma in IBD may contribute to intervention approaches that challenge and overcome stigma and foster resilience to stigma in those impacted, as have been successfully developed in other health conditions such as HIV/AIDS, mental illness, leprosy, tuberculosis, and epilepsy [26]. Qualitative research is well positioned for developing an understanding of the personal, contextualised, and nuanced causes, impacts of, and responses to stigma in those with IBD [27]. An emerging qualitative literature has begun to explore the lived experiences of stigma in IBD [23,28,29,30,31]. However, given the impact of stigma on wide-ranging aspects of life, the concept is also apparent within broader accounts of illness experiences in IBD where stigma was not the primary focus. This work seeks to build upon previous quantitative and mixed-methods reviews of stigma in IBD [19,20,25] by drawing together qualitative first-hand accounts from individuals across a range of cultural contexts, ages, symptom severity, and disease presentation to develop a unified account of the experience of stigma in IBD from the perspective of individuals living with the disease.

### Aim

This qualitative research synthesis employs a rigorous method for seeking greater meaning about the lived experiences of stigma and IBD through analysis, synthesis, and interpretation of a wider body of existing qualitative literature [32]. This review aims to not only systematically identify and summarise findings from previous literature but to develop novel interpretations and insights, providing an original framework of understanding stigma that is broad enough to enable findings to be generalised across individuals with IBD, yet focussed enough to provide contextually relevant theoretical insight [33,34].

Research question: What are the lived experiences of stigma in individuals with inflammatory bowel disease?

## 2. Materials and Methods

This review was carried out as a qualitative research synthesis [32] which aimed to identify, synthesise, and interpret the wider body of existing lived experience research to provide a new in-depth framework of understanding health-related stigma in individuals with IBD. The review was reported using the ENTREQ qualitative research statement [35].

### 2.1. Search Strategy

A systematic literature search was conducted by E.J. in May 2020. Five electronic databases (Medline, PsycINFO, Web of Science Core Collection, Scopus, and CINAHL) were purposively selected to retrieve articles within the fields of psychology, health, and nursing. Search terms are outlined in Table 1. Additional searches were conducted using citation tracking, manually searching reference lists of relevant articles, and forward citation searching. Database alerts were reviewed until May 2021 and identified five subsequently published articles for inclusion.

### 2.2. Study Eligibility

Studies were included if the article was (i) published in English language; (ii) published in a peer reviewed journal; (iii) primary research; (iv) qualitative research, or mixed-methods research if qualitative data were reported distinctly (i.e., studies which generate understanding of subjective experience using an interpretative framework, as defined by Noblit and Hare [36]); (v) study participants had a diagnosis of IBD, including Crohn’s disease or ulcerative colitis; (vi) study addressed a domain of stigma, as proposed by Weiss et al. [14]; (vii) reported findings were evidenced with participant quotes. There were no restrictions on age of study participants or publication date. Studies were excluded for (i) inclusion of other chronic illness; (ii) focus on a discrete aspect of IBD not universally experienced (e.g., surgery and stoma).

### 2.3. Literature Screening

**Figure 1:**
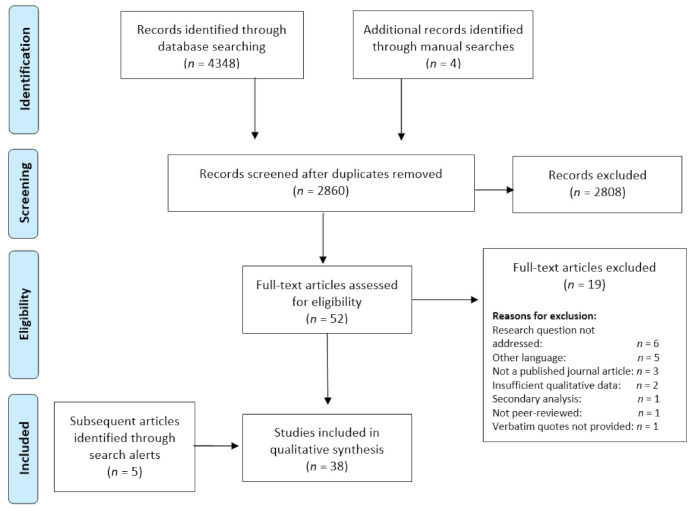
PRISMA flow diagram of the selection process. Adapted from the Preferred Reporting Items for Systematic Review and Meta-analyses (PRISMA) flow diagram [37].

Articles were collated and duplicates removed. The remaining articles (*n* = 2860) were screened by title and abstract, with 52 selected for full-text screening against the eligibility criteria. Thirty-eight studies were eligible for inclusions in analysis. The screening process is summarised in Figure 1.

### 2.4. Study Characteristics

The 38 studies included in the review were published from 1996 to 2021. Fifteen studies were conducted in the United Kingdom, five in Canada, five in Sweden, four in the United States, two in New Zealand, two in China, and one each in Hong Kong, Spain, Turkey, and Malta, and one in both the UK and Australia. A total of 1225 participants with an age range of 7–83 years were included. Thirty-one studies included an adult population, five included adults and adolescents, and two included children and adolescents. Seven studies focused on CD only, five on UC only and 26 studies included people with both CD and UC. Twenty-nine studies used individual interviews, one used focus group interviews, four combined individual and focus group interviews, one used video diaries and focus groups, one used interviews, friendship maps, and photographs, one used journal logs, and one used an online survey.

### 2.5. Quality Assessment

Quality of the included studies was assessed though the Critical Appraisal Skills Programme [38] CASP Qualitative Checklist, a suitable tool for examining quality across a range of qualitative methodologies [35]. All studies were reviewed independently by E.J. and K.M. who conferred findings. All included studies met all of the criteria in the CASP Checklist with the following exceptions (see Supplementary Table S1). Justification for methodological approach was not explicit in one study [39]. Consideration of the relationship between the researcher and the participants was unclear in 14 studies [29,39,40,41,42,43,44,45,46,47,48,49,50,51]. In one study ethical consideration was not clear [42]. One study provided ethical consideration but did not state if ethical approval was obtained [52]. Two studies did not provide a full description of analysis [30,39] and one did not provide a justification for analytic approach [53]. Despite these considerations, an appropriate level of quality was achieved across all studies and thus none were excluded on quality grounds.

### 2.6. Data Extraction

General characteristics were extracted to provide an overview of studies, consisting of authors and date of publication; study location; study population and number; aim of study; method of data collection; method of data analysis; key results or themes (reported in Table 2). Relevant data was then extracted from participant quotes and authors interpretations in the results. This approach allowed for analysis of rich accounts of original data alongside a triple hermeneutic approach which takes account of context to aid understanding [32].

### 2.7. Data Analysis, Synthesis, and Interpretation

Examination of the data moved through a cyclical process involving description, analysis, synthesis, and interpretation [32]. Descriptive comparisons of characteristics and findings across studies were used to compare and contrast study narratives as they sat alongside one another. Next, a detailed line by line coding of the data was conducted to note initial impressions of meaning. These codes were examined together to develop first order themes which described key dimensions of stigma occurring across studies. From this template, patterns were identified and examined across data to gain deeper insight into how the studies were positioned in relation to each other. Initial descriptive themes were drawn together into composite second order themes. These were constructed through an iterative process of moving between the original data, coding notes, and thematic frameworks to explore commonalities and differences across the studies. A matrix was used to record first and second order themes and their prevalence across studies.

In the final stages, themes were compared and condensed to generate third order themes. Here, the researchers sought to move away from aggregation, towards a novel interpretative understanding of the underlying processes and mechanisms related to the lived experiences of stigma in IBD. This was achieved through a process of careful consideration and reframing of underlying ideas and concepts, alongside revisiting original data. Across all stages of the analytic process, the authors were mindful to stay true to the voices of participants included in the synthesis. The final thematic structure was developed by K.M, supported by continual discussion, reflection, and checking with E.J. and A.D.

## 3. Results

The synthesis resulted in the development of two core categories of themes (see Table 3). ‘The IBD journey’ reflects the changing ways participants felt about themselves as a person living with IBD and the varying impact stigma has had on their sense of self. ‘A need to be understood’ describes the impact of IBD on the feeling of integration and connection within their social world. These themes are situated within an overarching concept ‘Feeling of Otherness’: feeling of difference and alienation from their lives and those around them. The distribution of themes across included papers are presented in Table 4. All quotations are drawn from study participants, unless labelled as an ‘author comment’.

### 3.1. The IBD Journey

This category of themes relates to the shifting ways in which having IBD impacted on individual’s self-identity. The disease became a central part of life, not only influencing how others viewed them but also how they viewed themselves. Narratives describe the ongoing personal journeys following a diagnosis of IBD. At times people felt the IBD label overshadowed and diminished them. Feelings of loss and shame accompanied an understanding of the self as a damaged and lesser version of a former self. Individuals described a process of re-building and recovery as they came to accept IBD as part of the self and sought a new sense of meaning, value, and self-worth.

#### 3.1.1. Reduced to a Label

This theme describes the experience of being defined primarily by a disease label. The IBD label was forefront in individuals lives, branding them as different. Participants felt IBD overshadowed all other aspects of self, impacting on other’s view of them and how they interacted with them as a person. This undermined their individuality and weakened their sense of being a whole person, giving the impression of being reduced to “just becoming identified by a stomach problem” [60]. Resentment arose when other people enabled the disease to eclipse the individual in this way:

“[The] consultant gave me a [national support group] leaflet and said, ‘You should get in touch because they’re very helpful for people like you.’ I remember walking out of the room thinking, ‘people like me?’ What the hell is he talking about? It took me ages to work out why that rubbed me up the wrong way. He was separating me out from the crowd and putting me in a box that went, ‘people like you over there. You’re not here, you’re over there’.”

[23]

Some adopted the label of IBD as a central focus of their own self-identity, it came to define how they identified themselves as a person. They viewed themselves as being the disease. This was a devaluing experience, diminishing their sense of self and making them feel like less of a person:

“It made me feel like it’s not colitis and Andrea, it’s Andrea with colitis, ya know, I was always putting it like at the front of my mind when I shouldn’t have been, I should have been thinking of myself more.” [47]“I can’t live like this … I don’t want to be a disease; I just want to be me.”

[66]

#### 3.1.2. The Spoiled Self

This theme illustrates the division between being ‘healthy’ and being ‘ill’. People compared themselves to their peers, positioning themselves as different and inferior. There was also a struggle to reconcile this aspect of selfhood with who they had understood themselves to be prior to having the disease. With this experience of alienation from the former self and others came the idea of being “damaged in some way” [43]. Value laden judgements were entrenched in the descriptions of the self as an unhealthy, diseased person who was tarnished by IBD. This provoked feelings of shame, guilt, and inadequacy:

“You just feel like you are different…you’re not a healthy person anymore. I just feel like an unhealthy person, so it just makes me feel like…I’m not good enough in a way.”

[56]

“I doubted myself, I doubted my value, I doubted my worth, I doubted my capability.”

[71]

Being unable to fulfil daily school, work, and family roles was related across accounts. Individuals reflected on feeling incapable and burdensome because they were unable to meet their own and other people’s expectations:

“I hate that I can’t handle having a full time job and having my kids. I don’t feel like I’m living up to the standards that I want for wife and mom, that’s one of my biggest struggles.”

[64]

Experiencing physical symptoms of IBD or side-effects from medications served as a reminder of the disease, evoking a feeling of being damaged. Individuals expressed concerns that visible marks of the disease such as weight change, scars, and skin sores would make them “look sickly” [49] and highlight what they felt was an ugly and unattractive illness to others. Internal experiences such as fatigue and pain also prompted internal dialogue of the self as a diseased person. Well intentioned expressions of sympathy or pity further compounded divisions by projecting images of sickness and fragility upon the person:

“My parents have never made any demands on me. I think this is negative. My mum felt pity about me. She has been coddling me all the time. I became more and more sad.”

[52]

#### 3.1.3. Re-Building the Self

This theme is about an ongoing process of re-building and recovery after a diagnosis of IBD. Narratives described a significant shift from the person individuals identified as prior to having IBD to who they felt they were after they received the diagnosis. People spoke of moving between striving to “regain normality” (author comment) [68] and acceptance of IBD as part of who they were. Some began to incorporate the life-long disease into their self-identity:

“It’s not going to go away, better it stays part of me, but I don’t want it to define me.”

[53]

Acceptance of IBD was especially hard for those who expressed loss of an integral part of the self and fought against what they felt was a lesser, sullied version of themselves:

“I don’t want to be considered “unhealthy.” What do I want? Empathy? Support? A visible symptom? I wouldn’t say no to any of them, but what do I really want? I just want me back.”; “I stand there facing the mirror. Not recognizing its reflection. Not wanting to recognize it. It’s not me. This is not me. Who am I kidding? This is what you have become. This is what’s left of you. Is this really me now?”

[63]

Accounts spoke of drawing on personal strengths and sources of private and professional support to re-build self-worth. Some rejected ideas of being a devalued person, showing defiance in the face of others’ negative or pitying attitudes. Others observed that discrimination and intolerance they faced helped them grow into a stronger person. In reconstructing the self, new aspects of the self were uncovered from which individuals regained mastery and derived value. In many accounts self-worth was gained from being able to channel the experience of living with IBD into being able to show compassion and support to others:

“I’ve helped a lot of people with Crohn’s and connected with them. I think maybe that’s what I’m here for. To help people dealing with the same thing…”

[64]

### 3.2. A Need to Be Understood

Woven throughout this category is the need to be understood. Accounts conveyed a strong need to be seen by others: for others in their lives to not only recognise but also make sense of their experiences of living with IBD. Yet individuals’ true experiences and perception of who they were as a person with IBD were often either hidden from or misunderstood by those around them. Accounts conveyed a lack of belonging to the wider social sphere, leaving people feeling isolated and marginalised. However, when individuals felt safe enough to be truly authentic with others, being understood was a liberating experience which fostered social connection.

#### 3.2.1. A Shameful Secret

Having an unseen disease meant others were not aware of and so could not understand the daily struggles individuals with IBD experienced, unless explicitly told. However, individuals lacked safe spaces in which they could reveal this hidden disease. This reluctance to fully disclose seemed to arise from a perception of disgust surrounding IBD as a disease. There was an idea that the nature of IBD being associated with the bowels and faeces made it a dirty disease, with many expressing revulsion at the nature of what they felt to be “gross and disgusting” [39] symptoms. Having IBD made people feel unclean and ashamed, driving them to limit what they shared or to conceal the disease altogether to avoid anticipated negative judgements from others:

“It comes from me and how I feel about [colitis]; I think it’s disgusting, horrible, and smelly—going to the toilet all the time and seeing all this gunk and blood and mucus. I think it’s disgusting, so I guess if people knew the full extent of what I see every day, they would think the same.”

[23]

Strategies such as diet restriction, staying close to toilets, and carrying spare clothes were employed to hide symptoms, even when these were detrimental to health. Others avoided situations where them having IBD would be uncovered:

“When I have to use the bathroom in the house of someone who is not a member of my family, I worry that the people there are thinking ‘look at him, coming to my house to constantly use my WC’, so I look for other alternatives because I don’t want people to think I’m dirty or weird’.”

[45]

Social rules relating to the distastefulness of discussing bowel habits were also raised. There was the impression that “some turn their noses up though when you talk about poo and wee and blood and stuff” [60]. This gave people the sense that they did not have permission from others to voice their experiences of IBD.

Living with the perpetual threat of being exposed as dirty and disgusting loomed over those who felt that IBD was a shameful secret. This was further exacerbated by the sporadic and unpredictable nature of symptoms, which made going out feel like “going into the unknown” [47]. Participants accounts conveyed anxiety, tension, and exhaustion underlying this ongoing concealment:

“I can’t tell you, talking about it is terrible because when I’m ill I don’t go out, I can’t face it. Whenever you go to use public toilets you’re never in a contained area. I can’t cope because of what everybody will think.”

[57]

#### 3.2.2. Knowing Is Not Understanding

This theme draws a distinction between knowing that somebody has IBD and really understanding the impact it has on their lives. As one person said “I think they don’t understand. They know I have this disease but they don’t know how it affects me.” [59]. A lack of awareness about IBD made it challenging and effortful to explain the disease to others. Accounts highlighted that varying misconceptions and prejudices about the nature of IBD created a further barrier to understanding, preventing people from receiving the recognition, validation, and support they craved. Some felt their experiences of having IBD were dismissed, minimised, or trivialised by those who did not recognise the enduring nature and severity of the disease. Others felt discredited and blamed for a problem that was perceived to be self-inflicted. Some accounts spoke of a fear that others believed they would be tainted or contaminated by coming into contact with the disease: “they say ‘Oh my God can I catch it off you?’” [55]. Individuals voiced a feeling of being devalued and condemned by these views, provoking intense reactions of frustration, pain, and loneliness:

“a prejudice of sorts that they have against me is when they say… come on, it’s just your stomach… they can’t understand, they go to the toilet like once every other day… ahh… I can’t keep it inside me… I just need to put up with it, so… it’s actually hard [tears start coming].”

[70]

“No one in my life understands what I am going through and strangers judge you and think you are contagious or disgusting.’’

[50]

Misconceptions were felt especially keenly when they came from close family members or healthcare professionals. It seemed that it was particularly damaging to feel misunderstood by those who were either part of one’s closest social circle or were expected to fulfil a caring role and show a greater level of understanding, empathy and compassion:

“When I first came out of hospital the last time my nan just called it a dicky tummy and I had IBS, so I felt pretty upset about that because that made me feel really like they didn’t understand what I went through, and they should’ve because they’re my family.”

[47]

“Once I was admitted to casualty with acute pain and still he [the doctor] told me that nothing was wrong. In the meantime I took tranquillizers and he [the doctor] told me to continue taking them since I was having panic attacks. but I knew that something was wrong... I began to doubt whether my family believed me or not and I would fill up with anger.”

[69]

#### 3.2.3. Social Estrangement

This theme describes the isolation accompanying social estrangement. The physical symptoms of IBD in combination with the stigma surrounding the disease disrupted how individuals were situated in their world, eroding a sense of belonging. Management of socially unacceptable and fluctuating physical symptoms and needs, especially around food and use of toilet facilities, severely constrained engagement in daily living. In restricting the ability to fully participate, having the disease made people feel excluded from these parts of their life. Shame about having what was felt to be a dirty and unclean disease provoked a sense of not being entitled to inhabit the same space. Feelings of loss and regret emerged from disconnection from parts of life which would ordinarily foster feelings of unity and shared identity:

“It’s just the way the ethnic community is and with food and obviously food is a big part of the culture… everything is based around food, weddings are based around food, you go into people’s houses, it’s all about bringing as much food as you can and that’s what entertaining is… It is a different culture and just, you know, you will stand out… because you’re not eating.”

[54]

Individuals felt ostracised for having symptoms which contravened societal expectations of normality and acceptability, including different diets, poor bowel control, loud or badly smelling bowel movements, taking medication, and use of feeding tubes. Some spoke of being “isolated and ridiculed” [49] by peers or family, who teased and bullied them. This was particularly apparent in accounts from young people. Even when overt social rejection was not directly experienced, there was strong fear that others may still be “*mentally backing away*” [23] because they are thinking badly of them:

“They don’t necessarily have to react badly, but if I think that they’re thinking something then…”

[55]

This feeling of exclusion was at times exacerbated by misplaced attempts of adaptation and support:

“At work they assigned me to places where I was on my own, so that I could easily go to the bathroom. In that way I would not hinder the work production.”

[69]

Comfort was sought by retreating to the safety of familiar surroundings. There was a pessimism underlying this, an idea that it was futile to try and be part of a social world where others either could not or would not accept or understand you. Although intended to serve as a protective factor, withdrawal created further barriers to social integration, inhibiting development of deeper bonds and leaving people feeling even more trapped and alone:

“People don’t get it, and I don’t expect them to, I guess. But, sometimes it’s easier just to not deal with it, and just stay in our little section, our little corner, on the farm, and be comfortable.”

[64]

“Relationships and friendships have suffered a great deal. I’ve found that my circle of friends and family members have very little understanding of IBD. Because of this I feel isolated and mostly keep to myself in my spare time.”

[50]

#### 3.2.4. The Freedom of Authenticity

There was tension in narratives between wanting people to understand and yet finding it too difficult, embarrassing, or shameful to be fully authentic and reveal the realities of living with IBD. Despite a strong need to feel heard, validated, and supported, many still felt driven by lack of understanding and fear of judgement or blame to hide this part of themselves:

“I’ve wondered if it would help if he [partner] knew a little more. But I don’t know, I don’t want to say all these symptoms, so many of them are gross. [If I communicated more] maybe he would be more empathic.”

[64]

“When I could not tell (I had Crohn’s disease), I felt really stressed. I also wanted them (my friends) to understand why I could not travel with them. However, I could not tell the truth.”

[67]

There was a palpable sense of relief when people were able to overcome this struggle and not only share their true experiences but be their true selves. Being authentic was liberating and empowering. As one person noted: “You kind of have that freedom once you tell people. You don’t have to hide it anymore” [30]. It was easier for people to let their guard down in the context of trusted and supportive friendships or family environments, where they felt safe enough to offer this level of openness. The intimacy of full disclosure helped individuals feel understood by and re-connected with others:

“I think if anything those friends have become closer because I think they feel I’ve shared a lot more with them now because they know so much about it and they know how hard it’s been, they’ve been there in the hospital and wherever else and they’re still with me when I feel terrible so they see it face to face.”

[47]

Empathy and support were also gained from others who also had a diagnosis of IBD and therefore had similar lived experiences. When somebody shared this experience, many of the barriers to authenticity were broken down, allowing shared understanding. This was affirming and supported individuals to feel reconnected:

“I met someone at work who also had UC. And it was good to talk to someone who understands exactly my situation. It made me feel less alone with this problem.”

[62]

### 3.3. Overarching Concept: Feeling of Otherness

The two core themes are nested within an overarching concept of otherness. Otherness was a lens through which individuals experienced their lives and interacted with the world around them. It was a subtle and insidious form of estrangement and devaluing which impacted across individuals lives. Otherness influenced individual’s engagement in the world around them and permeated the language and behaviour of friends, family, colleagues, and healthcare professionals, separating those with IBD out as being different. It also shaped how individuals with IBD viewed themselves, resulting in a feeling of disconnection between the ‘ill’ self and a former ‘healthy’ self. Yet this stigmatising experience of otherness was variable rather than static. Across time and situations, individuals fluctuated between the separation of otherness and inclusion of integration. This was influenced by disease activity, previous experience of coping, quality of support networks, others’ actions and words, expectations of others’ views, awareness, and knowledge of IBD, and the social situation. A feeling of integration was derived by re-building a valued sense of self and through authentic social connection to others who were aware of, understood, and accepted the individual for who they were. Central to this was acceptance of an inclusion of IBD in the individual’s internal and external world. Whilst the experience of otherness was marked by profound isolation and exclusion, the experience of integration was accompanied by freedom and empowerment.

## 4. Discussion

This synthesis draws together findings from 38 studies describing lived experiences to provide insight into when, how, and why stigma impacts those with IBD. The two core themes incorporated the interconnected personal and social journeys that individuals with IBD embarked on following diagnosis. Stigma was a common experience within these journeys, involving intrapersonal, interpersonal, and community level processes of exclusion, separation, withdrawal, isolation, and judgement [72]. Themes within ‘the IBD journey’ explore the diminishing experience of being ‘reduced to a label’, the shame accompanying an understanding of the self with IBD as ‘the spoiled self’, and a process of recovery and self-acceptance within ‘re-building the self’. Themes within ‘a need to be understood’ consider the distressing experience of feeling driven to keep a ‘a shameful secret’, the painful impact of feeling misunderstood in ‘knowing is not understanding’, the sense of ‘social estrangement’ arising from a lack of social belonging, and finally in ‘the freedom of authenticity’ the comfort gained from authentic connection with others.

Stigma had broad health and social impacts on wellbeing, self-worth, identity, quality of life, social relationships, healthcare interactions, illness management, working life, and education. In line with the multi-faceted conceptualisation of stigma [17], a distinction can be drawn between the experience of enacted, perceived, and internalised stigma. Perceived stigma has been reported to commonly occur in IBD [19,20] and has an adverse impact on mental health and wellbeing [73,74]. Perceived stigma was highly prevalent throughout this synthesis. The themes ‘knowing is not understanding’ and ‘social estrangement’ describe a perception that others hold negative views around disgust, blame, condemnation, or dismissal towards people with IBD, due to the unclear cause and nature of the disease relating to the bowels. This view resulted in intense emotional reactions of anxiety, frustration, pain, and loneliness, indicating that perceived stigma may have a similarly harmful impact on psychological functioning as in other long-term conditions such as HIV/AIDS and mental health [75,76]. Enacted stigma is reported as less common than other forms of stigma in IBD [19,20]. Although teasing, bullying, and being ridiculed was evident within the theme ‘social estrangement’, such forms of explicit discrimination were less frequent and mostly limited to young people’s accounts. However, more subtle forms of discrimination were evident within the ‘spoiled self’, which speaks of an image of sickness or fragility being projected upon individuals by others treating them as less capable, misplaced attempts of adaptation and support, and unwelcome demonstrations of pity. Thus, less overt and intentional discrimination may be more widespread in IBD than previously considered.

Enacted and perceived stigma were reported from peers, co-workers, friends, family, healthcare providers, and the general public. However, within ‘knowing is not understanding’ it can be seen that concern about those closest to them demonstrating prejudice or holding stigmatising views appeared most damaging. In the face of feeling stigmatised, people frequently cited close social networks as crucial sources of support. Yet when family and friends were unable or unwilling to provide empathy or themselves showed stigma, there was an increased sense of stigma burden which further impeded wellbeing and placed a heavy strain on those important relationships. This supports the suggestion that kinship stigma (negative or discriminatory attitudes towards individuals by family members and their closest relationships) can have an intense emotional impact [23,28]. Additionally, perceived or enacted stigma from healthcare professionals was also experienced as being particularly distressing. This suggests that the detrimental effect of stigma is greatest from those in positions expected to be supportive, empathic, and understanding, whether that is family, friends or healthcare providers.

Mild levels of internalised stigma were reported previously in IBD [19,20] and have been linked with the poorest psychological outcomes of the three stigma domains [20], especially when accompanied by shame [19]. In this synthesis, internalised stigma was strongly apparent across the themes ‘reduced to a label’, ‘the spoiled self’, and ‘a shameful secret’, where accounts conveyed the shameful experience of viewing the self with IBD as diminished and dirty. Internalised stigma has been reported as more prevalent in IBD during an active disease phase [20]. Findings from this synthesis indicate this may be due to physical symptoms serving as a reminder of IBD and evoking internal dialogue of the self as dirty and damaged. The socio-cognitive model provides a framework for understanding the process by which some people came to hold negative self-views: through stereotype-awareness, stereotype-agreement, and self-concurrence [77]. Accounts in this review show that individuals were aware of and sometimes held negative societal stereotypes about dirtiness and disgust surrounding bowel functions before they received an IBD diagnosis. Awareness was further heightened following diagnosis, with individuals assuming that negative views would be shared by others. Those who internalised negative stereotypes viewed them as legitimate and felt they applied to them as somebody with a bowel disease, diminishing their self-esteem, and making acceptance challenging.

The overarching concept ‘feeling of otherness’ highlights that, rather than a static, binary experience, individuals moved back-and-forth along a continuum, ranging from the excluding experience of otherness, to the inclusive experience of integration. Integration involved intrapersonal and interpersonal processes of freedom, empowerment, empathy, validation, and belonging, giving rise to feelings of relief, acceptance, and being valued. Studies have differentially reported that length of disease duration is associated with a reduction, increase, or no change in levels of stigma [19]. These mixed findings may reflect the fluctuating nature of stigma in IBD, which varies across physical, social, and health contexts within which the individual is situated. Flare-ups in particular brought an increased susceptibility to stigma as they evoked a self-concept of a ‘spoiled self’ and increased fear of others noticing symptoms as considered in ‘a shameful secret’. Movement towards integration was supported through the process of psychological adjustment to IBD apparent within ‘re-building the self’ and reconnecting with valued others through illness disclosure as explored within ‘the freedom of authenticity’. Drawing on experience of adaptive coping further strengthened these forms of stigma resistance during more challenging times.

This synthesis suggests that psychological adjustment to IBD plays a key role in protecting individuals from the detrimental effects of stigma. Positive adjustment to a chronic illness is a transitional process involving acceptance of the illness, incorporation of it into the existing sense of self, and sometimes self-growth [78]. In the current synthesis, being diagnosed with IBD caused individuals to feel ‘reduced to a label’, devaluing them and consuming their self-identity. In viewing themselves as a ‘spoiled self’, they felt tarnished and alienated from their former self. However, by accepting and incorporating IBD into their identity, as a part rather than the whole, they were able to embark on the process of ‘re-building the self’ and recover feelings of self-worth. For some, having IBD also uncovered new aspects of the self from which they derived value. As with other fluctuating conditions [78], living with ongoing unpredictability and uncertainty made acceptance especially challenging. This aligns with the “push and pull” phenomenon identified by Kemp and colleagues [8], with the fluctuation of disruption to life trapping individuals in a cycle of pushing for normality but being pulled back from this by the disease. Thus, acceptance of IBD may mean acceptance of multiple, shifting versions of “normality”. This process may be more difficult for those who experience internalised stigma, as those who felt shame about what was understood to be a ‘spoiled self’ struggled with acceptance.

Within this synthesis, stigma provoked a number of unhelpful avoidance-based coping strategies. The themes ‘a shameful secret’ and ‘social estrangement’ examine a complex convergence of fear that others would not accept or understand the disease, the taboo nature of the symptoms, worry that others would think they were unclean due to the nature of the symptoms relating to the bowels, and a feeling of being dirty due to having IBD, all of which prompted avoidance and social withdrawal. These cycles of avoidant behaviour exacerbated feelings of difference and further trapped and disempowered individuals, thereby fuelling anxiety and low mood. Whilst such maladaptive coping strategies have been linked to poorer psychological health for those with IBD, use of more adaptive coping styles are associated with higher wellbeing and quality of life [79]. Within this synthesis, individuals who were able to draw on previous experiences of adaptive coping were more able to cope with the demands of living with a stigmatised illness. Within ‘freedom of authenticity’ this took the form of seeking social support and within ‘re-building the self’ in positively reappraising the situation. The ability to identify and flexibly apply a range of adaptive coping strategies may support resilience to the impacts of stigma [80].

A challenging decision for many individuals diagnosed with an invisible illness is how and when to disclose to others. ‘A shameful secret’ highlights that feelings of shame associated with believing that IBD was a dirty, disgusting disease that others would also view negatively compelled some to employ strategies that were detrimental to their own health and wellbeing to facilitate concealment. Hiding the disease inhibited individual’s ability to build effective support networks, a known outcome of concealment in other stigmatising health conditions [24,81]. It was also difficult and effortful to conceal IBD from others due to ‘flare ups’ and noticeable impacts on life such as missing social events, leaving people feeling vulnerable to ‘involuntary’ disclosure [82]. Disclosure was made more challenging by the lack of public knowledge and understanding about IBD [83] and societal norms which dictate that the bowels and faeces are not something that should be discussed in social settings [24]. However, within ‘freedom of authenticity’ we see that some individuals felt able to disclose their illness experiences and support their social network to understand this new part of their life. This resulted in feelings of liberation and empowerment, supporting their recovery from social disconnection. Such voluntary and planned disclosure of stigmatised diseases is recognised as a crucially important factor in patient health and wellbeing [82]. However, it was not only disclosure of the diagnostic label but full disclosure of the impact of this disease which seemed key in feeling understood and connected to others.

### 4.1. Implications and Recommendations

There may be multiple pathways through which perceived, enacted, and internalised stigma leads to negative outcomes for individuals with IBD. As such, a multi-level approach targeting stigma at the intrapersonal, interpersonal, and structural level may be required [84]. Psychological support could be used to help individuals with IBD cope with stigma. Cognitive-behavioural therapy (CBT) has been found to reduce internalised stigma and improve psychological and social functioning for people with stigmatised mental health conditions [26]. CBT-based interventions in IBD could target unhelpful avoidance or withdrawal strategies, support use of more adaptive coping strategies, enable re-appraisal of self-stigmatising beliefs, and facilitate individual’s adjustment to living with IBD. The disparity between perceived and enacted stigma suggests individuals may overestimate the threat of stigma from others and raises the potential of re-examination of threat-appraisals. Navigating the complexities of the disclosure process and helping individuals with IBD decide when, to whom, and how to disclose their diagnosis will also be important [82]. Supportive disclosure may also have the additional benefit of improving interpersonal interactions and therefore reducing stigmatising attitudes of others [84]. Acceptance-based CBT approaches such as Acceptance and Commitment Therapy and Mindfulness-Based Cognitive Therapy may also address unhelpful avoidance-based coping strategies as well as fostering greater self-compassion and acceptance of living with IBD [85,86]. For some, a systemic approach may be beneficial in establishing helpful connections, coping, and adaptation to IBD within individuals’ family systems [87]. Given the fluctuating and context-dependent nature of stigma in IBD, psychological interventions could usefully be employed at the time of diagnosis, to foster skills for building stigma resistance and reduce the likelihood of more significant psychological difficulties developing. Developing resource-light interventions is important given that psychology provision within gastroenterology services is often under-resourced. High acceptability of electronic psychological interventions has been reported in those with IBD [88], indicating potential for less costly computerised interventions. Individuals in the current synthesis reported that support from others with lived experience was valuable and research has shown that meeting others with the same or similar conditions can provide information and emotional support [89], thus group-based formats may be valuable and cost-effective. Additionally, healthcare settings could increase advertising and engagement in online peer support networks established by charities for people with IBD (e.g., cicra.org, crohnscolitisfoundation.org, crohnsandcolitis.org.uk).

Education-based interventions could target stigmatised views in the community. Within this synthesis, the development of stigma related to IBD appears to stem from societal ideas that faeces and defecation is an inherently dirty and disgusting process, which should not be discussed or in any way be made public, almost that engaging in this vital bodily function should be denied and viewed like a dirty secret. Educational interventions could be used to refute negative stereotypes surrounding the bowels by presenting information contradicting stereotypes or by creating cognitive dissonance, which have been effective in changing attitudes and behaviours towards individuals with HIV/AIDs and mental health problems [84]. Participants in this synthesis believed there was a general lack of awareness and understanding about IBD, a viewpoint which is illustrated in studies demonstrating a lack of knowledge of IBD in the general public [90]. Increased understanding of IBD would likely make disclosure easier as well as reducing stigma and prejudice views, as has been shown in relation to mental health difficulties [91,92]. As well as targeting negative stereotypes, educational campaigns could discuss how best to support individuals with IBD. As misplaced efforts to provide support can be experienced as stigmatising, this may reduce unintentional enacted stigma. Supporting family members and important others to understand IBD and the experience of living with it could be especially important, allowing them to perform a key supportive role. Involvement of experts by experience in development and delivery of educational material will be key, as including contact with someone with the stigmatised condition improves the effectiveness of awareness-based interventions [84]. The ‘it takes guts’ campaign (ittakesguts.org.uk) showcases important work which has already begun in providing a platform for such conversations. Healthcare providers may also benefit from further education about IBD from a lived experience perspective, as individuals in the synthesis found it especially difficult when healthcare providers were less empathic than expected. Understanding of the psychosocial impact of IBD in particular may be limited within GPs [93] and the impact of IBD on daily functioning has also been found to be underestimated in gastroenterologists [94]. Communication training may support busy medical professionals consider effective ways of talking about IBD with patients in clinic, to help prevent unintentionally indicating a lack of interest or recognition of the impact of IBD on their patients’ lives. Finally, rather than challenging stigma, resources could be used to support parents and teachers to promote positive schemas about bowel functioning during early development. Similar early stigma interventions have been found to be effective in promoting positive childhood attitudes in the context of mental health [95]. Early work to prevent the development of taboos and shame around defecation may be more effective than later attempting to dismantle negative stereotypical views which have become more fixed. This may also protect against internalised stigma by reducing the likelihood of stereotype endorsement following a diagnosis of IBD. Reducing the taboo around bowel functioning and the internalised stigma that can come with IBD has the potential to alleviate some of the burden of living with IBD and improve patient’s health-related quality of life.

Findings from this synthesis highlight important avenues for future exploration. There is a need to investigate the occurrence of subtle discriminatory practices towards those with IBD and the impact this form of enacted stigma may have on individuals’ lives. It would be useful to examine the differential occurrence, experience, and impact of perceived and enacted stigma from different sources to further explore the concept of kinship stigma and whether this extends to healthcare professionals. Research could examine what supports individuals to develop resistance to stigma in IBD, in particular resistance to internalised stigma and a reduction in perceived stigma. Finally, the review highlighted a paucity of research sampling children or adolescents. Yet the prevalence of paediatric onset IBD is rising [96], with approximately one quarter of IBD cases presenting before adulthood [97]. Reports in this synthesis of teasing or bullying related to IBD were more apparent amongst paediatric populations, suggesting there may be distinctions in the type of stigma experienced by young people. Stigma may be particularly impactful in this group, as adolescence is a crucial age for social development where peer judgments have a strong influence on far reaching aspects of self-hood, including perceptions of social and personal worth [98]. Increased appraisals of IBD stigma have been shown to be associated with reduced perceptions of social belongingness, resulting in higher rates of depressive symptoms in an adolescent population [99]. It could therefore be valuable to better understand the experience of stigma in this population.

### 4.2. Strengths and Limitations

One of the key strengths of this review is the large sample of participants accounts that were drawn upon to provide a rich and detailed narrative of the lived experiences of stigma in individuals with IBD. Despite a reasonable level of diversity amongst included participants in terms of gender, ethnicity, sexuality, age, disease length, and disease activity, findings demonstrate many commonalities in the experiences of stigma and the mechanisms through which it operates. However, findings must be viewed within the context of the study limitations. First, findings were drawn from published quotations and author interpretations from included studies and therefore analysis did not involve examination of the original raw data. Second, only including studies published in English language means that evidence published in other languages was not incorporated. Third, although three studies were conducted in China and Hong Kong, the majority of studies included a Western sample. Given the rise of IBD in newly industrialised countries [3], it will be important to understand any differences in the experience of stigma for those with IBD across different cultural contexts.

## 5. Conclusions

This synthesis highlighted the complexity of stigma and IBD; different forms of stigma are apparent, each with the potential to impact on the individual’s physical and mental wellbeing in different ways, including low mood, social withdrawal, and reduced self-esteem. Perceived and internalised stigma were more commonly reported than enacted stigma. Whilst some of the adolescent population reported teasing and bullying, for adults enacted stigma was often viewed as misplaced attempts to be supportive or a lack of awareness and understanding. Kinship stigma, and stigma from healthcare professionals, appeared to be the most challenging for individuals due to them having greater expectations of understanding and empathy within these valued relationships. Stigma was not a binary experience; individuals fluctuated along the continuum between feeling stigmatised and othered and feeling integrated and connected dependent on disease activity and different social and environmental contexts. Psychological adjustment to IBD, the use of adaptive coping strategies, and illness disclosure were important protective factors. Development and implementation of interventions to support individuals who experience IBD-related stigma and to foster stigma resistance will be important. Interventions could also seek to reduce IBD-related stigma by providing education for families, social networks, and healthcare providers on the psychosocial impact of IBD, and by implementing stigma reduction interventions at a community level.

## Figures and Tables

**Table 1:** Electronic database search terms.

IBD	Experience	Qualitative
“Inflammatory Bowel Disease” “Colitis, Ulcerative” “Crohn Disease” colitis[Title/Abstract] crohn*[Title/Abstract]	Experience*[Text Word] Perspective*[Text Word] “living with”[Title/Abstract] life[Title/Abstract] influence[Title/Abstract] impact[Title/Abstract] effect[Title/Abstract]	Interview*[All Fields] qualitative[All Fields] themes[All Fields]

Note: Search terms within each construct (IBD, experience, and qualitative) were combined with *OR* whilst search terms between each construct were combined with *AND.* The * was used to search for all forms of the word that start with the same letters.

**Table 2:** Attributes of included studies.

Author and Date	Location of Research	Population and Number	Aim of Study	Data Collection	Data Analysis *	Results/Findings
Alexakis et al. (2015) [54]	UK	Young people with IBD from BME groups (16–24 yrs) *n* = 20	To identify and characterise the experiences (positive and negative) and difficulties faced by young IBD patients from BME communities.	Semi-structured interviews.	Thematic analysis.	Four themes: Culture and religion Parents, families, and the wider community Education Healthcare services and support
Barned et al. (2016) [40]	Canada	Children and adolescents with IBD (10–17 yrs) *n* = 25	To determine how children and adolescents with IBD go about deciding if and when to tell others about their illness.	Semi-structured interviews.	Thematic analysis.	Three themes: To disclose or conceal: making the decision When to tell: factors influencing disclosure decisions - Gaining adequate knowledge about one’s illness - Severity of the illness Challenges of IBD disclosure: the reactions of others
Brydolf and Segesten (1996) [52]	Sweden	Adolescents and young adults with UC (11–31 yrs) *n* = 28	To gain a deeper understanding of the adolescents’ experiences of how it felt to live with UC.	Interviews.	Constant comparative method for grounded theory.	Eight categories: Alienation Reduced living space Support Lack of support Confidence in self Disbelief in self Role identification as a child/patient Role identification as an adult
Carter et al. (2020) [55]	UK	Young people with IBD (14–25 yrs) *n* = 31	Exploring stigma and disclosure in young people with IBD.	Interviews, friendship maps, and photographs.	Interpretive description.	Three themes: To tell or not to tell Controlling the flow: the who, when, what, and how of telling Reactions and responses to telling: anticipated and actual
Cho et al. (2018) [56]	Canada	Young adults with IBD (18–30 yrs) *n* = 21	To identify the health-related quality of life needs of young adults with IBD.	Interviews.	Constant comparative method.	Four broad categories of needs: Psychosocial needs Informational needs Self-advocacy needs Daily living needs
Cooper et al. (2010) [57]	UK	Adults with IBD (30–40 yrs) *n* = 24	To explore beliefs about personal control and self-management of IBD.	Semi-structured interviews.	Systematic framework analysis.	One main theme: Reconciliation of the self in IBD Three sub-themes: Living with uncertainties and contradictions I’ve got it or it’s got me Evaluating images of me and health
Czuber-Dochan et al. (2012) [58]	UK	Adults with IBD (27–80 yrs) *n* = 46	To explore fatigue, its impact on daily life and the strategies used to ameliorate the symptom, as described by people with IBD.	Focus group interviews.	Inductive thematic framework.	Five themes: The experience of fatigue Causes of fatigue Managing fatigue Consequences of fatigue Seeking support
Czuber-Dochan et al. (2020) [59]	UK	Adults with IBD (17–63 yrs) *n* = 28	To address unmet needs regarding psychosocial aspects of food, eating, and drinking in IBD.	Semi-structured interviews.	Colaizzi’s framework.	Five themes: Personal experience of relationship between IBD and food Managing diet to control IBD and its symptoms Impact of food-related issues on everyday life Acceptance and normalisation of food and its impact in IBD Sources of information and support
Daniel (2002) [43]	Canada	Young adults with IBD (18–24 yrs) *n* = 5	To expand awareness and enhance empirical understanding of the young adult IBD patient’s perspective of living with IBD.	Semi-structured interviews.	Descriptive phenomenological method.	Themes in the study compared with King’s concepts in the Personal System, Interpersonal System and Social System
Demirtas (2021) [41]	Turkey	Adults with IBD (25–58 yrs) *n* = 25	To determine the life experiences of patients with IBD.	Semi-structured interviews.	Phenomenological method.	Three main themes: A flare phase of the disease A remission period Coping behaviours
Devlen et al. (2014) [44]	USA	Adult with IBD (20–59 yrs) *n* = 27	To describe the impacts of IBD from a patient perspective.	Focus groups and individual interviews.	Grounded theory.	A conceptual model of impacts including: Immediate impacts Lifestyle impacts Impacts of daily activities Impacts on social and leisure activities Treatment impacts Psychological impacts Impacts on relationships
Dibley et al. (2014) [29]	UK	Gay and lesbian people (GLP) with IBD (27–54 yrs) *n* = 22	To explore the parallels between coming out about sexual identity and IBD in order to compare GLP IBD-related concerns with those in the non-gay IBD community and to identify the social and psychological aspects of IBD in GLP.	Semi-structured interviews.	Pragmatic thematic analysis.	Four central themes: GL sexual activity Receiving health care IBD and GL life Identity and coming out
Dibley et al. (2018) [23]	UK	Adults with IBD (23–78 yrs) *n* = 40	To explore the lived experience of stigma in IBD.	Unstructured interviews.	Interpretive hermeneutic phenomenological analysis.	Three constitutive patterns: Being in and out of control Relationships and social support Mastery and mediation
Dibley et al. (2019) [28]	UK	Adults with IBD (21 – 64 yrs) *n* = 18	An exploration of the experience and meaning of kinship stigma in people with IBD.	Unstructured interviews.	Interpretive hermeneutic phenomenological analysis.	Three relational themes: Being Visible/Becoming Invisible Being the Disease/Having the Disease Amplification, Suffering, and Loss Constitutive Pattern: Lacking Acknowledgment/Being Acknowledged
Dudley-Brown (1996) [42]	Hong Kong	Adults with UC (30–58 yrs) *n* = 3	To describe real life experiences of patients with UC.	Semi-structured interviews.	Phenomenological analysis.	Five themes: Uncertainty surrounding the length of time between exacerbations of symptoms Fear and humiliation accompanying stool incontinence The desperate need to find successful treatment and return to normal life The profound effect of family life, social life, and work The feeling of being controlled by the disease
Frohlich (2014) [30]	USA	Adults with IBD (20–56 yrs) *n* = 14	To understand how people with IBD experience stigma because of their disease.	Semi-structured interviews.	Identification of common themes.	Six potentially stigmatising illness sites: Initial diagnosis Romantic relationships Work and school Surgery Medicine Overt stigma
García-Sanjuán, et al. (2017) [45]	Spain	Adults with CD (25–83 yrs) *n* = 19	To understand the lived experience of CD.	Interviews.	Colaizzi’s framework.	Five themes: Self-protection against the unknown cause self-training Learning to live with CD Perceived losses associated to CD Relationship with others
Hall et al. (2005) [60]	UK	Adults with IBD (28–79 yrs) *n* = 31	To gain a better understanding of the perspectives and experiences of individuals with IBD and a poor quality of life.	Individual semi-structured interviews and focus groups.	Grounded theory.	Emergent core concept: ‘Health-related normality’
Jordan et al. (2017) [61]	UK	Adults with IBD (22–68 yrs) *n* = 25	To explore the experience of people with IBD and elevated symptoms of anxiety and low mood and the type of psychological help they would like.	Semi-structured interviews.	Template analysis.	Two themes related to anxiety: under performance; preventing an accident Two themes related to low mood: lack of understanding; stigma One main theme for type of psychological help desired: Expertise and understanding
Larsson et al. (2016) [62]	Sweden	Adults with IBD (29–63 yrs) *n* = 15	To investigate the specific disease-related stress in individuals with IBD, how they cope with this stress and what help is requested from the healthcare.	Interviews.	Content analysis.	Three central areas: Stress: disease-related stress and relations to others Coping: behavioural strategies, social strategies and emotional strategies Need for help or support: instrumental support and emotional support.
Lynch and Spence (2008) [46]	New Zealand	Adolescents and young adults with CD (16–21 yrs) *n* = 4	To explore how youth experience living with recently diagnosed CD.	Semi-structured interviews.	Thematic analysis.	Three themes: Stress as integral to living with CD The paradoxical relationship between fear and hope What helps and what hinders
Matini and Ogden (2016) [47]	UK	Adults with IBD (18–39 yrs) *n* = 22	To explore the notion of adaptation in patients with IBD.	Semi-structured interviews.	Thematic analysis.	Three core themes: Making sense of the illness Impact Feelings Overarching theme: Uncertainty
Mikocka-Walus et al. (2020) [53]	UK (*n* = 13) and Australia (*n* = 11)	Adults with IBD (20–70 yrs) *n* = 24	To explore the lived experience and healthcare needs of patients with IBD and mild-to-moderate comorbid anxiety and/or depression.	Semi-structured interviews and focus groups.	Thematic analysis (UK) and template analysis (Australia)	Three UK themes: Bidirectional relationship between IBD and mental health, Need for healthcare integration Lack of awareness about the disease Three Australia themes: The ‘vicious cycle’ of IBD and psychosocial health The need for biopsychosocial healthcare integration and The stigma of a hidden disease
Moore (2013) [63]	UK	Adult with UC (20 yrs) *n* = 1	To document the author’s lived experience while in the midst of an acute flare-up of UC.	Journal logs.	Autoethnography.	Reflection upon the influence of illness on an athletic body within a sporting subculture.
Mukherjee et al. (2002) [48]	UK	Parents with IBD (26–54 yrs) *n* = 24	To identify parents’ views on how IBD affects people in their parenting role; effects parents with IBD have noticed in their children; ways of dealing with any difficulties in parenting; support needed by parents with IBD.	Individual interviews and focus groups.	Framework approach.	Five main themes: Effects on parents Effects on children Ways of dealing with difficulties Support received from services Messages for service providers
Nicholas et al. (2007) [49]	Canada	Children and adolescents with IBD (7–19 yrs) *n* = 80	To understand the lived experience and elements of quality of life as depicted by children and adolescents with IBD.	Semi-structured interview.	Content analysis.	Five themes: Concerns relating to IBD symptoms and treatments Vulnerability and lack of control Perceiving the self negatively as different than peers Benefits of social support Personal resources in coping
Norton et al. (2012) [39]	USA	Adults with CD (18–75 yrs) *n* = 48	To understand the impact of CD on various aspects of daily life from the perspective of patients living with CD.	Video diaries and focus groups.	Descriptive summaries.	Impact of CD on various aspects of life: General impact of CD Relationship with provider Psychological impact of CD Social impact of CD Impact of CD on activities Impact of CD on professional life
Nutting and Grafsky (2017) [64]	USA	Five adult heterosexual couples with CD (28–40 yrs) *n* = 10	To understand how a partner’s diagnosis of CD is perceived to affect couple relationship functioning and satisfaction, as well as young adult life-cycle transitions.	Interviews (each partner interviewed individually).	Interpretive phenomenological analysis.	Four areas of experiences: Diagnosis Biopsychosocial wellbeing Relationship functioning and satisfaction Life-cycle transitions
Lesnovska et al. (2010) [65]	Sweden	Adults with CD (29–83 yrs) *n* = 11	To identify and describe the meaning of quality of life in patients with CD.	Interviews.	Grounded theory.	Five dominant themes: Self-image Confirmatory relations Powerlessness Attitude toward life Sense of well-being
Lesnovska et al. (2016) [66]	Sweden	Adults with IBD (29–83 yrs) *n* = 30	To describe how patients living with IBD experience critical incidents in daily life in relation to their disease and symptoms.	Interviews.	Critical incident technique (inductive).	Five categories: Losing bowel control Having a body that smells Being unable to meet own and others’ expectations Not being believed or seen Experiencing frustration due to side effects and ineffective treatment. One main area describing the overall result: The bowels rule life
Purc-Stephenson et al. (2014) [50]	Canada	Adults with IBD (18–62 yrs) *n* = 378	To explore the positive and negative changes patients with IBD have experienced since diagnosis.	Online survey.	Grounded theory.	Five themes related to positive changes: Interpersonal Relations, Personal Growth, Valuing Life, New Life Paths, and Spiritual Growth. Three themes related to negative changes: Freedom Restrictions, Psychological Side Effects, and Social Isolation
Richard et al. (2020) [51]	New Zealand	Adults with IBD (30–79 yrs) *n* = 18	To explore how adults living with IBD in rural New Zealand manage their condition and engage with healthcare providers.	Semi-structured interviews.	Thematic analysis.	Five constructs: Journey to confirming and accepting diagnosis Importance of the relationship with the healthcare team Support from others Learning how to manage IBD Care at a distance
Ruan et al. (2020) [67]	China	Adults with IBD (21–58 yrs) *n* = 16	To explore the experiences of body image changes in patients with IBD in China and to describe how those changes influence patients’ perception of body and self.	Semi-structured interviews.	Content analysis.	Six themes: Being a constrained person Being a flawed person Being a disliked person Being an alienated person Being a reconciled person Being a blessed person
Ruan and Zhou (2019) [68]	China	Adults with CD (19–68 yrs) *n* = 31	To explore the illness experiences of patients with CD in China and construct an interpretive understanding of these experiences from the perspective of the patients.	Interviews.	Grounded theory.	Four categories: Comparing Struggling Reflecting Realising One core category: Regaining normality
Sammut et al. (2015) [69]	Malta	Adults with UC (29–60 yrs) *n* = 10	To explore the experiences of adults living with UC.	Semi-structured interviews.	Interpretative phenomenological analysis.	Three super-ordinate themes: Living with physical discomfort Emotional turmoil in living the experience Social interactions
Saunders (2014) [31]	UK	Adults with IBD (18–29 yrs) *n* = 16	To explore how stigma is discursively constructed by young adults, with a focus on the moral underpinnings of the participants’ talk.	Semi-structured interviews.	Rhetorical discourse analysis.	Representations showed both felt stigma and enacted stigma; principally related to the perceived taboo surrounding the symptoms of their condition, which often led to the non-disclosure or concealment of the condition
Vejzovic et al. (2018) [70]	Sweden	Adolescents with UC (13–18 yrs) *n* = 7	To illuminate the meaning of children’s lived experience of UC.	Interviews.	Phenomenological hermeneutical method.	One main theme: Daily struggle to adapt and be perceived as normal Four subthemes: Being healthy despite the symptoms Being healthy despite being afraid Being healthy despite a sense of being different Being healthy despite needing support
Wilburn et al. (2017) [71]	UK	Adults with CD (25–68 yrs) *n* = 30	To understand how the lives of people with CD are affected.	Interviews.	Theoretical thematic analysis.	Thirteen main need themes: Nutrition, hygiene, continence, freedom from infection, security, self-esteem, role, attractiveness, relationships, intimacy, clear-mindedness, pleasure, and autonomy

* Data analysis is outlined as described in the original article. Note: BME = black and minority ethnic, IBD = inflammatory bowel disease, CD = Crohn’s disease, UC = ulcerative colitis.

**Table 3:** Categories, themes, and overarching concept.

Category	Theme	Overarching Concept
The IBD journey	Reduced to a label The spoiled self Re-building the self	Feeling of Otherness
A need to be understood	A shameful secret Knowing is not understanding Social estrangement The freedom of authenticity	

**Table 4:** Distribution of themes across studies included in the review.

	The IBD Journey			A Need to Be Understood			
Author and Date	Reduced to a Label	The Spoiled Self	Re-Building the Self	A Shameful Secret	Knowing Is Not Understanding	Social Estrangement	The Freedom of Authenticity
Alexakis et al. (2015) [54]		✓		✓	✓	✓	✓
Barned et al. (2016) [40]	✓	✓		✓	✓	✓	✓
Brydolf and Segesten (1996) [52]		✓	✓		✓	✓	✓
Carter et al. (2020) [55]	✓			✓	✓	✓	✓
Cho et al. (2018) [56]		✓		✓	✓	✓	
Cooper et al. (2010) [57]		✓	✓	✓	✓		
Czuber-Dochan et al. (2012) [58]	✓	✓	✓	✓	✓		✓
Czuber-Dochan et al. (2020) [59]	✓		✓		✓	✓	✓
Daniel (2002) [43]		✓	✓	✓	✓	✓	
Demitras (2021) [41]		✓		✓	✓	✓	
Devlen et al. (2014) [44]		✓	✓	✓	✓	✓	
Dibley et al. (2014) [29]		✓	✓	✓	✓	✓	✓
Dibley et al. (2018) [23]	✓	✓	✓	✓	✓	✓	✓
Dibley et al. (2020) [28]	✓	✓	✓	✓	✓	✓	✓
Dudley-Brown (1996) [42]		✓	✓	✓	✓	✓	
Frohlich (2014) [30]	✓	✓		✓	✓	✓	✓
García-Sanjuán et al. (2017) [45]		✓	✓	✓	✓	✓	✓
Hall et al. (2005) [60]	✓	✓	✓	✓	✓	✓	✓
Jordan et al. (2017) [61]		✓	✓	✓	✓	✓	✓
Larsson et al. (2016) [62]		✓	✓	✓			✓
Lynch and Spence (2008) [46]		✓	✓	✓	✓	✓	✓
Matini and Ogden (2016) [47]	✓	✓	✓		✓		✓
Mikocka-Walus et al. (2020) [53]	✓		✓	✓	✓	✓	
Moore (2013) [63]	✓	✓	✓	✓	✓	✓	✓
Mukherjee et al. (2002) [48]		✓		✓		✓	✓
Nicholas et al. (2007) [49]	✓	✓	✓	✓	✓	✓	✓
Norton et al. (2012) [39]		✓		✓	✓	✓	
Nutting and Grafsky (2017) [64]		✓	✓	✓	✓	✓	✓
Lesnovska et al. (2010) [65]	✓	✓	✓	✓		✓	✓
Lesnovska et al. (2016) [66]	✓	✓		✓	✓	✓	✓
Purc-Stephenson et al. (2014) [50]		✓	✓		✓	✓	
Richard et al. (2020) [51]			✓			✓	✓
Ruan et al. (2020) [67]		✓	✓	✓	✓	✓	✓
Ruan and Zhou (2019) [68]	✓	✓	✓	✓	✓	✓	✓
Sammut et al. (2015) [69]		✓			✓	✓	
Saunders (2014) [31]		✓		✓	✓	✓	✓
Vejzovic et al. (2018) [70]	✓	✓	✓	✓	✓	✓	✓
Wilburn et al. (2017) [71]		✓				✓	

## Data Availability

Data included in this synthesis is available from the originally published articles included, as listed in the reference list.

## Supplementary Materials

The following are available online at https://www.mdpi.com/article/10.3390/ijerph18158038/s1, Supplementary Table S1: CASP appraisal.
