# Supplementary material for: A Feeling of Otherness: A Qualitative Research Synthesis Exploring the Lived Experiences of Stigma in Individuals with Inflammatory Bowel Disease
Source: Int J Environ Res Public Health. 2021 Jul 29;18(15):8038. doi: 10.3390/ijerph18158038 (PMC8345596; doi:10.3390/ijerph18158038)
Supplement: Supplementary file 1 [file ijerph-18-08038-s001.zip › ijerph-1311467-supplementary.pdf]

Table S1. Critical Appraisal Skills Programme (2019) Qualitative Checklist for included articles.

	1. Statement of aims	2. Appropriate methodology	3. Appropriate design	4. Appropriate recruitment strategy	5. Data collection method justified and clear	6. Consideration of relationship b/tw researcher & ppts	7. Consideration of ethical issues	8. Rigorous data analysis	9. Clear statement of findings	10. Is the research valuable?
Alexakis et al. (2015) [54]	Yes	Yes	Yes	Yes	Yes	Yes	Yes	Yes	Yes	Yes
Barned et al. (2016) [40]	Yes	Yes	Yes	Yes	Yes	Can't tell.	Yes	Yes	Yes	Yes
Brydolf and Segesten (1996) [52]	Yes	Yes	Yes	Yes	Yes	Yes	Yes. Consideration given but ethics approval not stated.	Yes	Yes	Yes
Carter et al. (2020) [55]	Yes	Yes	Yes	Yes	Yes	Yes	Yes	Yes	Yes	Yes
Cho et al. (2018) [56]	Yes	Yes	Yes	Yes	Yes	Yes	Yes	Yes	Yes	Yes
Cooper et al. (2010) [57]	Yes	Yes	Yes	Yes	Yes	Yes	Yes	Yes	Yes	Yes
Czuber-Dochan et al. (2012) [58]	Yes	Yes	Yes	Yes	Yes	Yes	Yes	Yes	Yes	Yes
Czuber-Dochan et al. (2020) [59]	Yes	Yes	Yes	Yes	Yes	Yes	Yes	Yes	Yes	Yes
Daniel (2002) [43]	Yes	Yes	Yes	Yes	Yes	Can't tell.	Yes	Yes	Yes	Yes
Demirtas (2021) [41]	Yes	Yes	Yes	Yes	Yes	Can't tell.	Yes	Yes	Yes	Yes
Devlen et al. (2014) [44]	Yes	Yes	Yes	Yes	Yes	Can't tell.	Yes	Yes	Yes	Yes
Dibley et al. (2014) [29]	Yes	Yes	Yes	Yes	Yes	Can't tell.	Yes	Yes	Yes	Yes
Dibley et al. (2018) [23]	Yes	Yes	Yes	Yes.	Yes	Yes	Yes	Yes	Yes	Yes
Dibley et al. (2019) [28]	Yes	Yes	Yes	Yes.	Yes	Yes	Yes	Yes	Yes	Yes
Dudley-Brown (1996) [42]	Yes	Yes	Yes	Yes	Yes	Can't tell.	Can't tell.	Yes	Yes	Yes
Frohlich (2014) [30]	Yes	Yes	Yes	Yes	Yes	Yes	Yes	Can't tell. In-depth description of the analysis process not provided.	Yes	Yes
García-Sanjuán, et al. (2017) [45]	Yes	Yes	Yes	Yes	Yes	Can't tell.	Yes	Yes	Yes	Yes
Hall et al. (2005) [60]	Yes	Yes	Yes	Yes	Yes	Yes.	Yes	Yes	Yes	Yes

Jordan et al. (2017) [61]	Yes	Yes	Yes	Yes	Yes	Yes.	Yes	Yes	Yes	Yes
Larsson et al. (2016) [62]	Yes	Yes	Yes	Yes	Yes	Yes	Yes	Yes	Yes	Yes
Lynch and Spence (2008) [46]	Yes	Yes	Yes	Yes.	Yes	Can't tell.	Yes	Yes	Yes	Yes
Matini and Ogden (2016) [47]	Yes	Yes	Yes	Yes	Yes	Can't tell.	Yes	Yes	Yes	Yes
Mikocka-Walus et al. (2020) [53]	Yes	Yes	Yes	Yes	Yes	Yes	Yes	No, divergent analytic methods for two parts of the data set not justified or explained.	Yes	Yes
Moore (2013) [63]	Yes	Yes	Yes.	Yes	Yes	Yes	Yes	Yes	Yes	Yes
Mukherjee et al. (2002) [48]	Yes	Yes	Yes	Yes	Yes	Can't tell.	Yes	Yes	Yes	Yes
Nicholas et al. (2007) [49]	Yes	Yes	Yes	Yes	Yes	Can't tell.	Yes	Yes	Yes	Yes
Norton et al. (2012) [39]	Yes	Yes	Yes.	Yes.	Can't tell. Justification for this approach not explicit.	Can't tell.	Yes	Can't tell. In-depth description of the analysis process not provided.	Yes	No
Nutting and Grafsky (2017) [64]	Yes	Yes	Yes	Yes	Yes	Yes	Yes	Yes	Yes	Yes
Lesnovska et al. (2010) [65]	Yes	Yes	Yes	Yes	Yes	Yes	Yes	Yes	Yes	Yes
Lesnovska et al. (2016) [66]	Yes	Yes	Yes	Yes	Yes	Yes	Yes	Yes	Yes	Yes
Purc-Stephenson et al. (2014) [50]	Yes	Yes	Yes	Yes	Yes	Can't tell.	Yes	Yes	Yes	Yes
Richard et al. (2020) [51]	Yes	Yes	Yes	Yes	Yes	Can't tell.	Yes	Yes	Yes	Yes
Ruan et al. (2020) [67]	Yes	Yes	Yes	Yes	Yes	Yes	Yes	Yes	Yes	Yes
Ruan and Zhou (2019) [68]	Yes	Yes	Yes	Yes	Yes	Yes	Yes	Yes	Yes	Yes
Sammut et al. (2015) [69]	Yes	Yes	Yes	Yes	Yes	Yes	Yes	Yes	Yes	Yes
Saunders (2014) [31]	Yes	Yes.	Yes	Yes	Yes	Yes	Yes	Yes	Yes	Yes
Vejzovic et al. (2018) [70]	Yes	Yes	Yes	Yes	Yes	Yes	Yes	Yes	Yes	Yes
Wilburn et al. (2017) [71]	Yes	Yes	Yes	Yes	Yes	Yes	Yes	Yes	Yes	Yes
